# Supplementary material for: Sex-specific single cell-level transcriptomic signatures of Rett syndrome disease progression
Source: Commun Biol. 2024 Oct 10;7:1292. doi: 10.1038/s42003-024-06990-0 (PMC11464704; doi:10.1038/s42003-024-06990-0)

Supplementary fig.1: Identification of significant DEGs using Limma, EdgeR and DESeq2 produced few common genes from male sn-RNA seq data

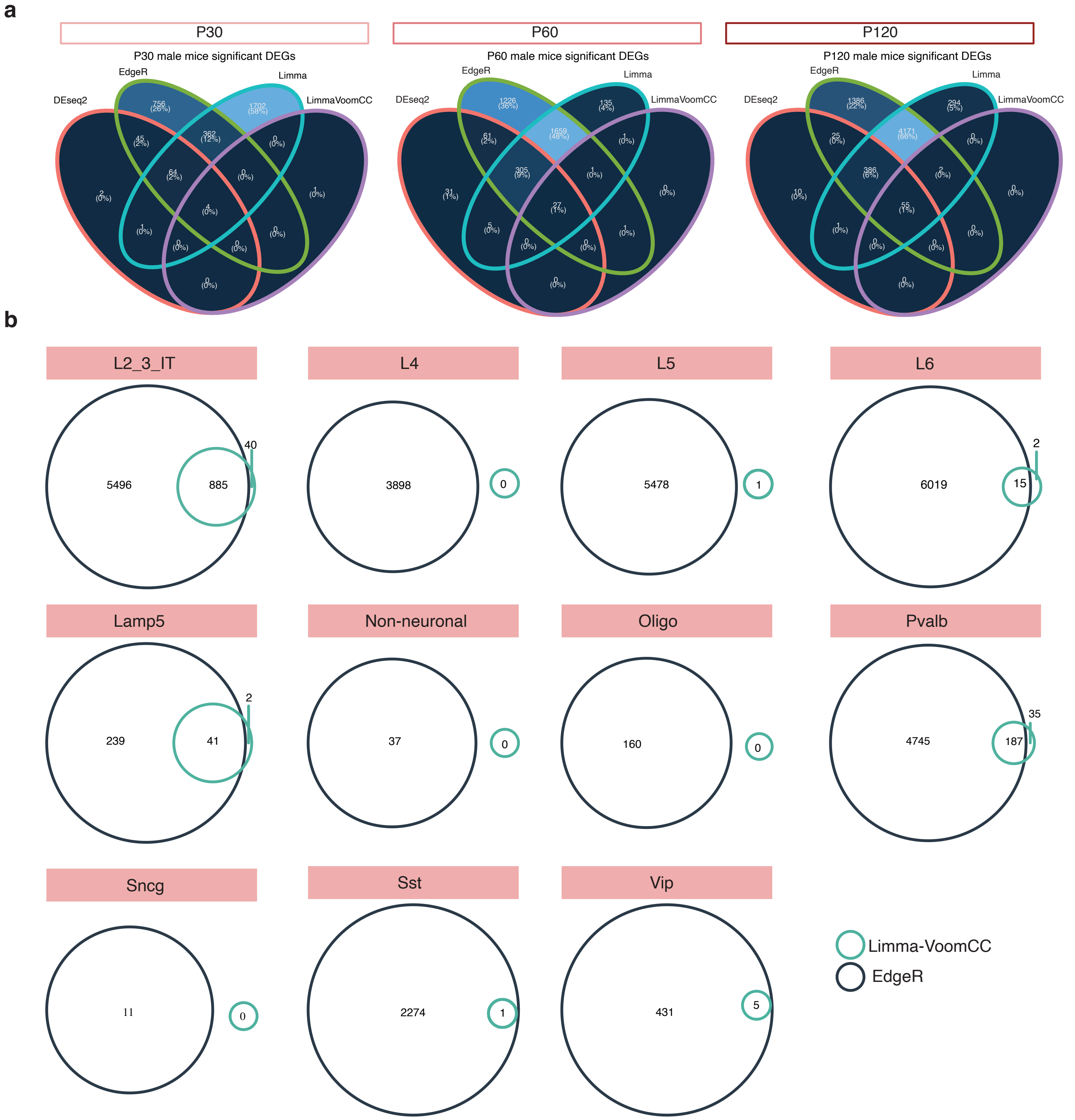

Supplementary fig.2: Line graph of the number of post-natal DEGs identified using DESingle from *Mecp2e1*$^{-/+}$ female and *Mecp2e1*$^{-/y}$ cortices

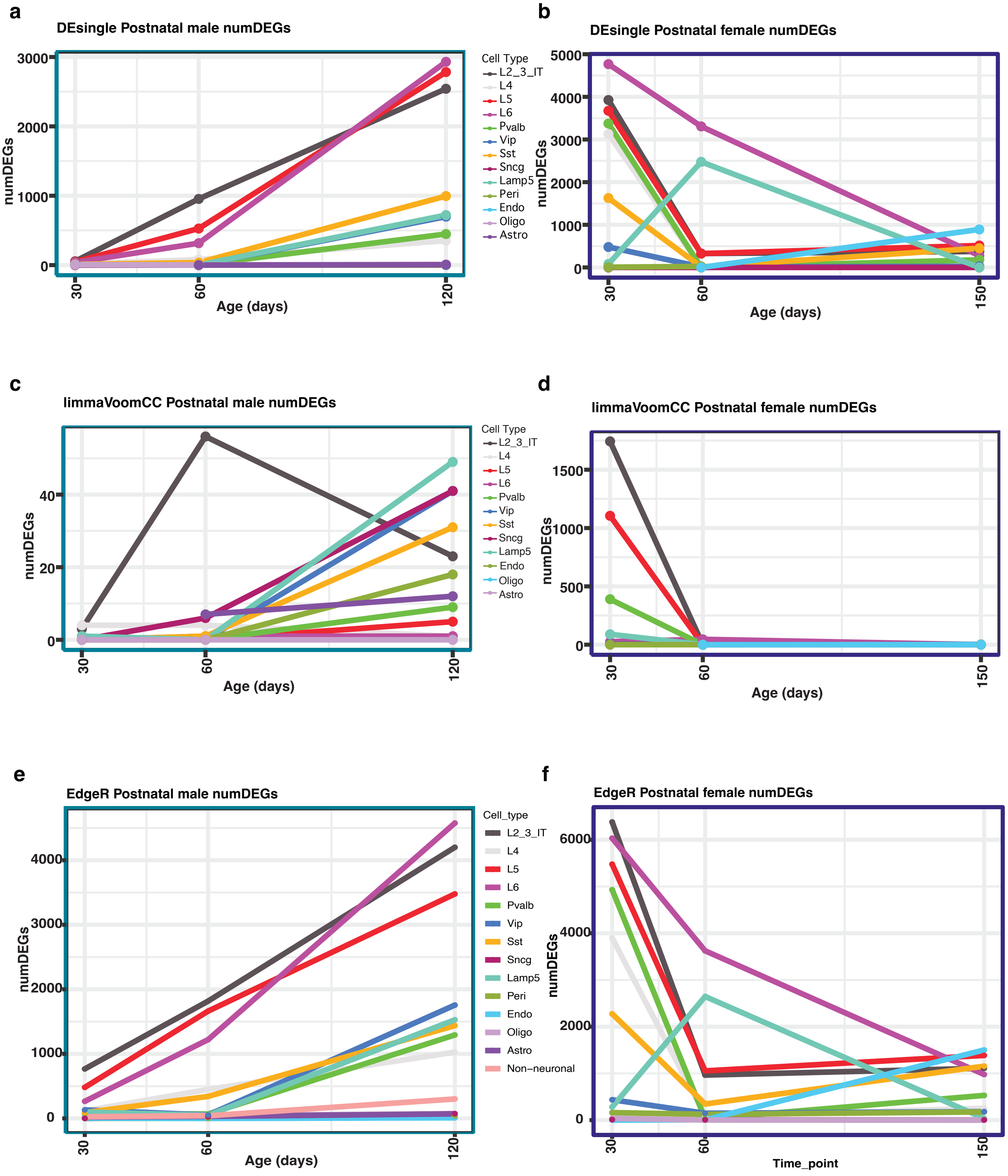

Supplementary fig.3: *Mecp2e1* mutant cortices have normal proportions of cortical cell types

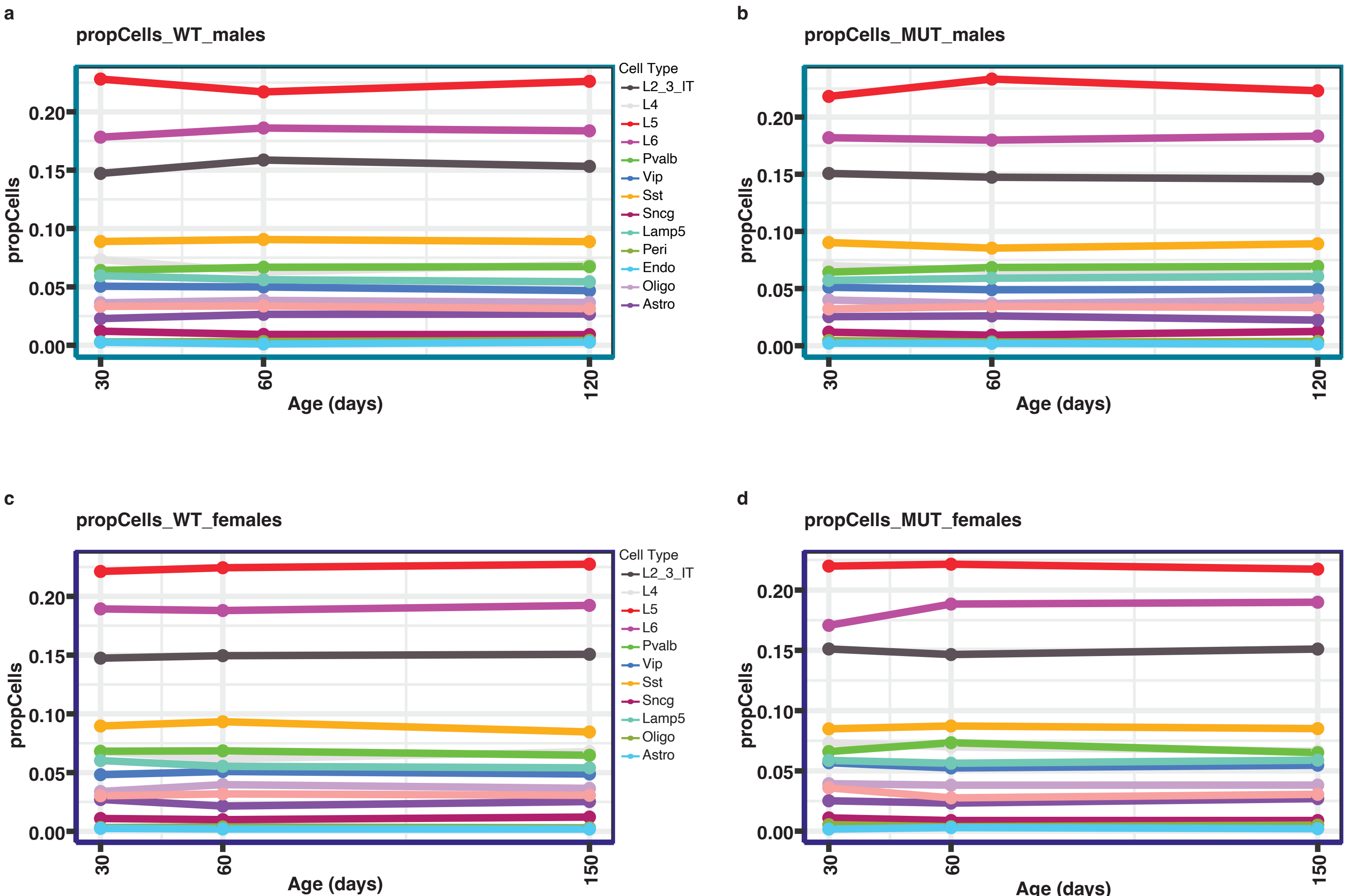

Supplementary fig.4: Cell clustering does not differ between sexes and *Mecp2* genotypes in postnatal cortex

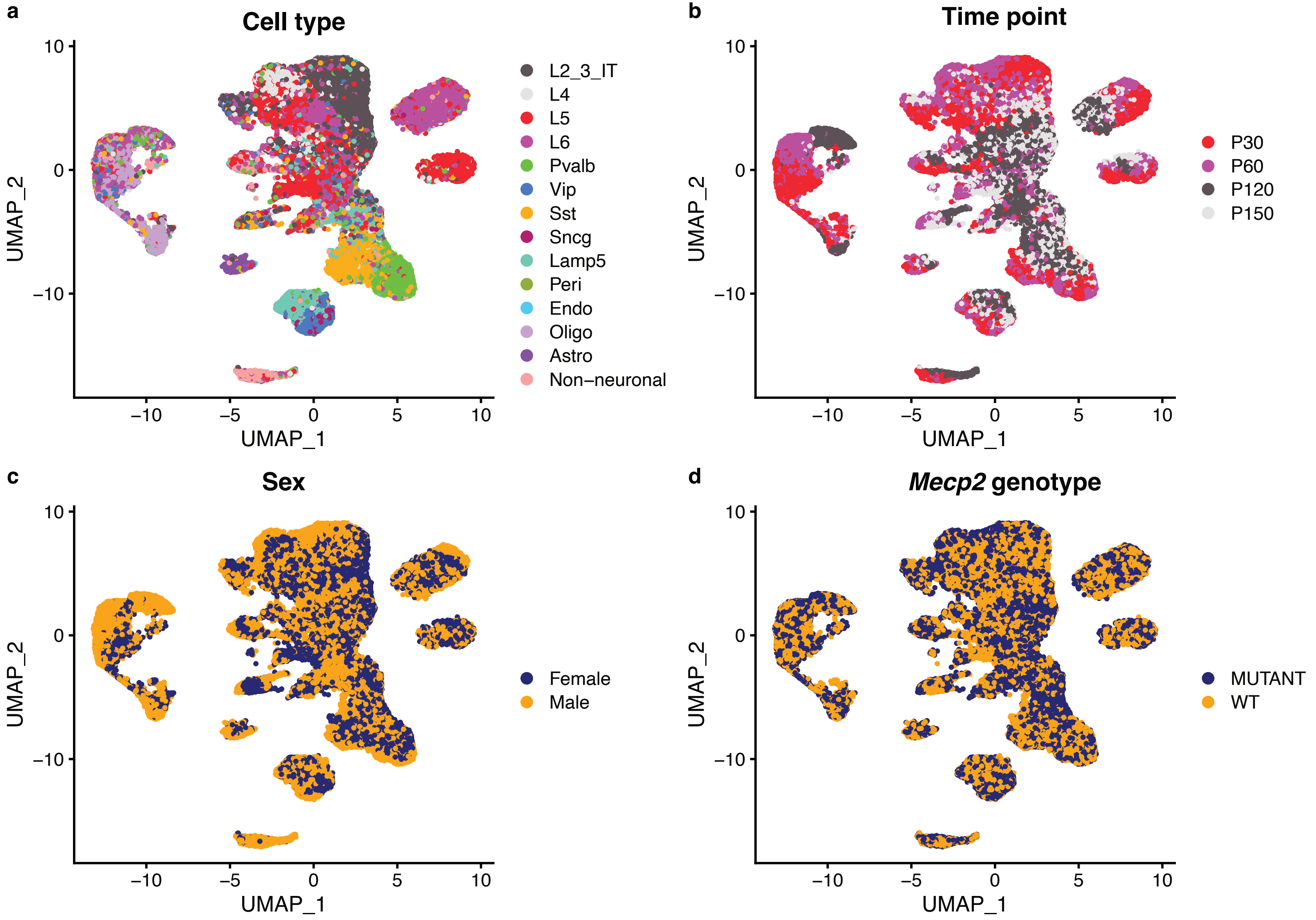

Supplementary fig.5: Replicate mouse brain samples have similar patterns of cell type-specific genes expressed in common

a

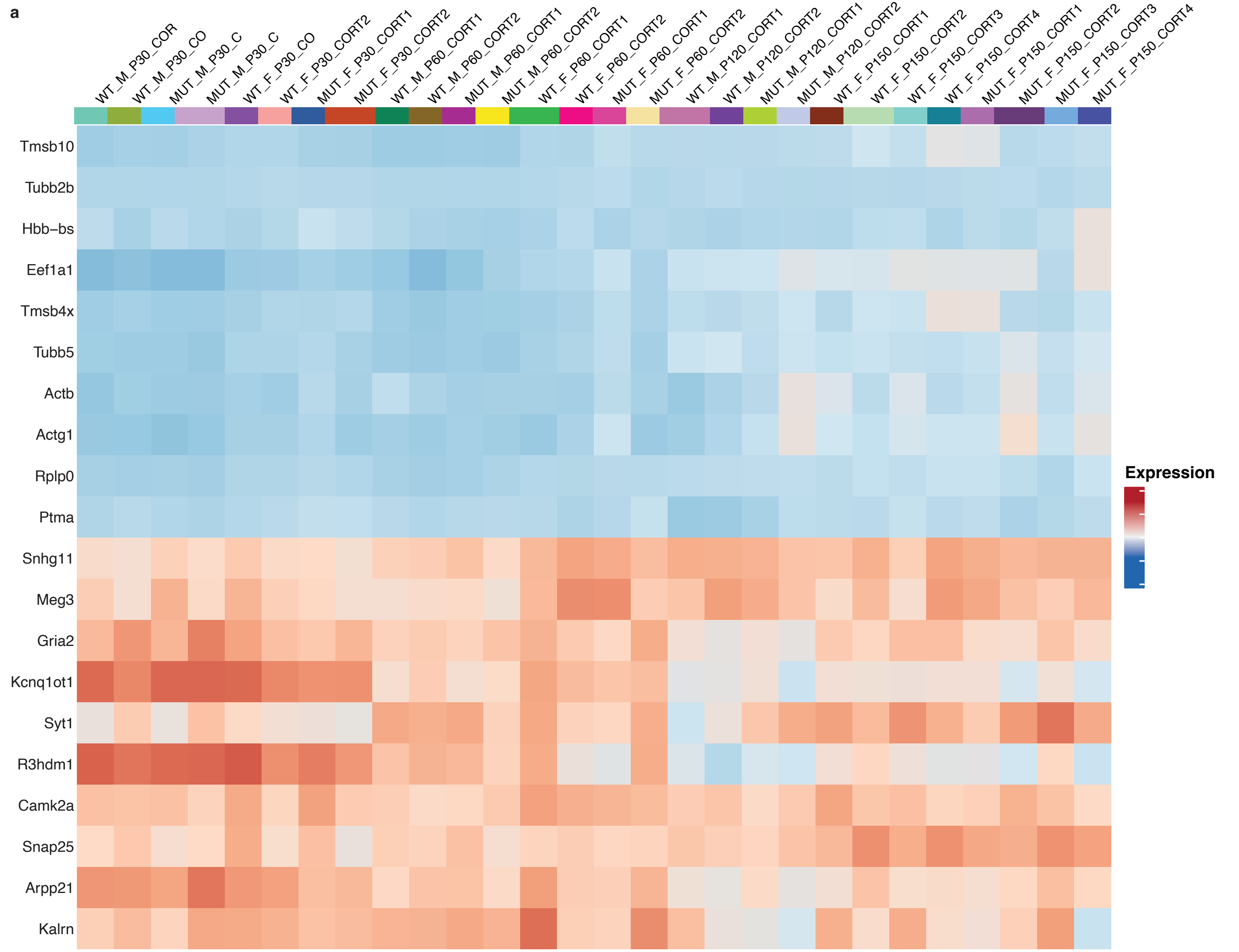

Supplementary fig.6: Differentially expressed genes are not correlated with gene length

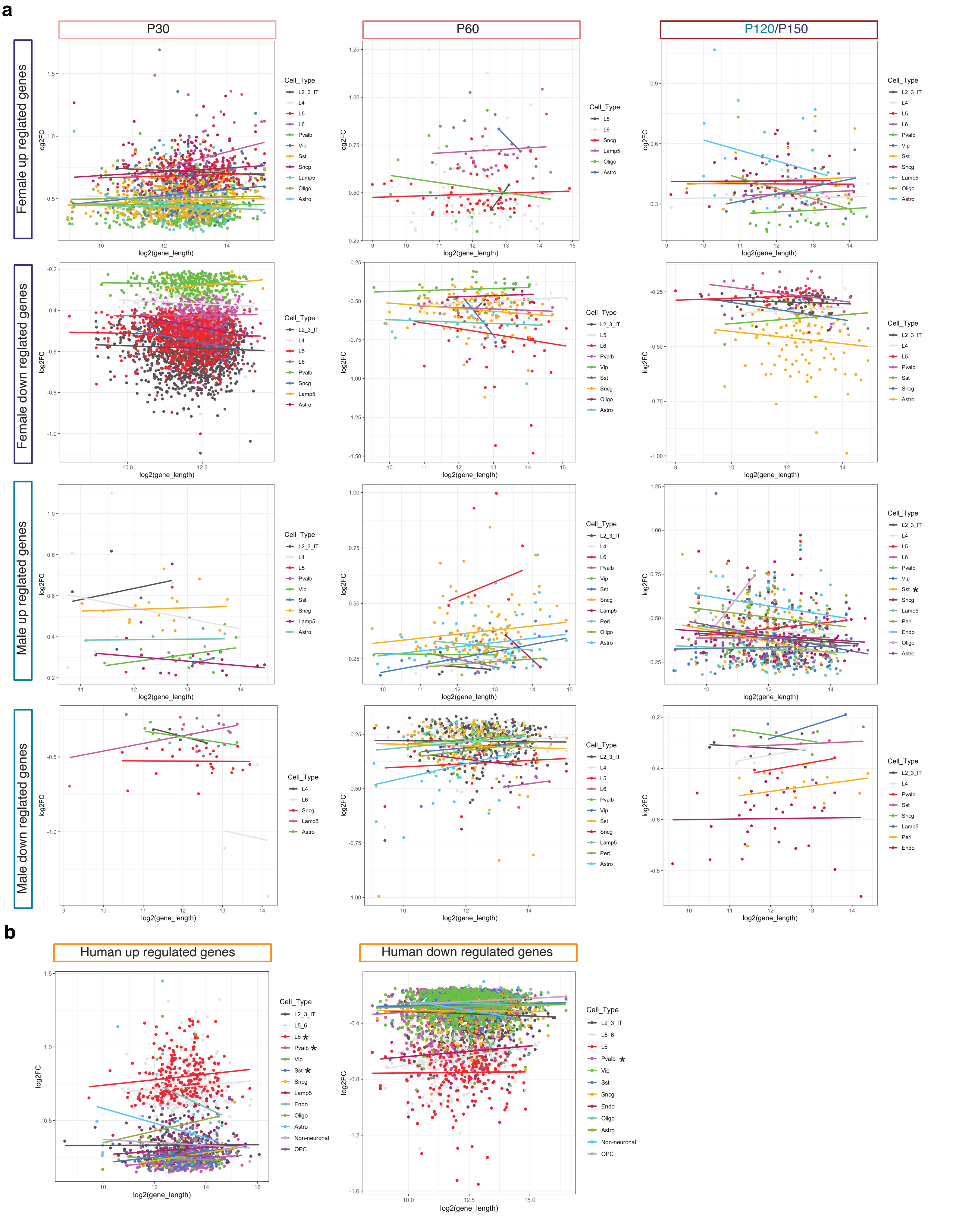

Supplementary Fig.7: Complete co-expression networks for each cell type in the mouse cortex and RTT relevant traits

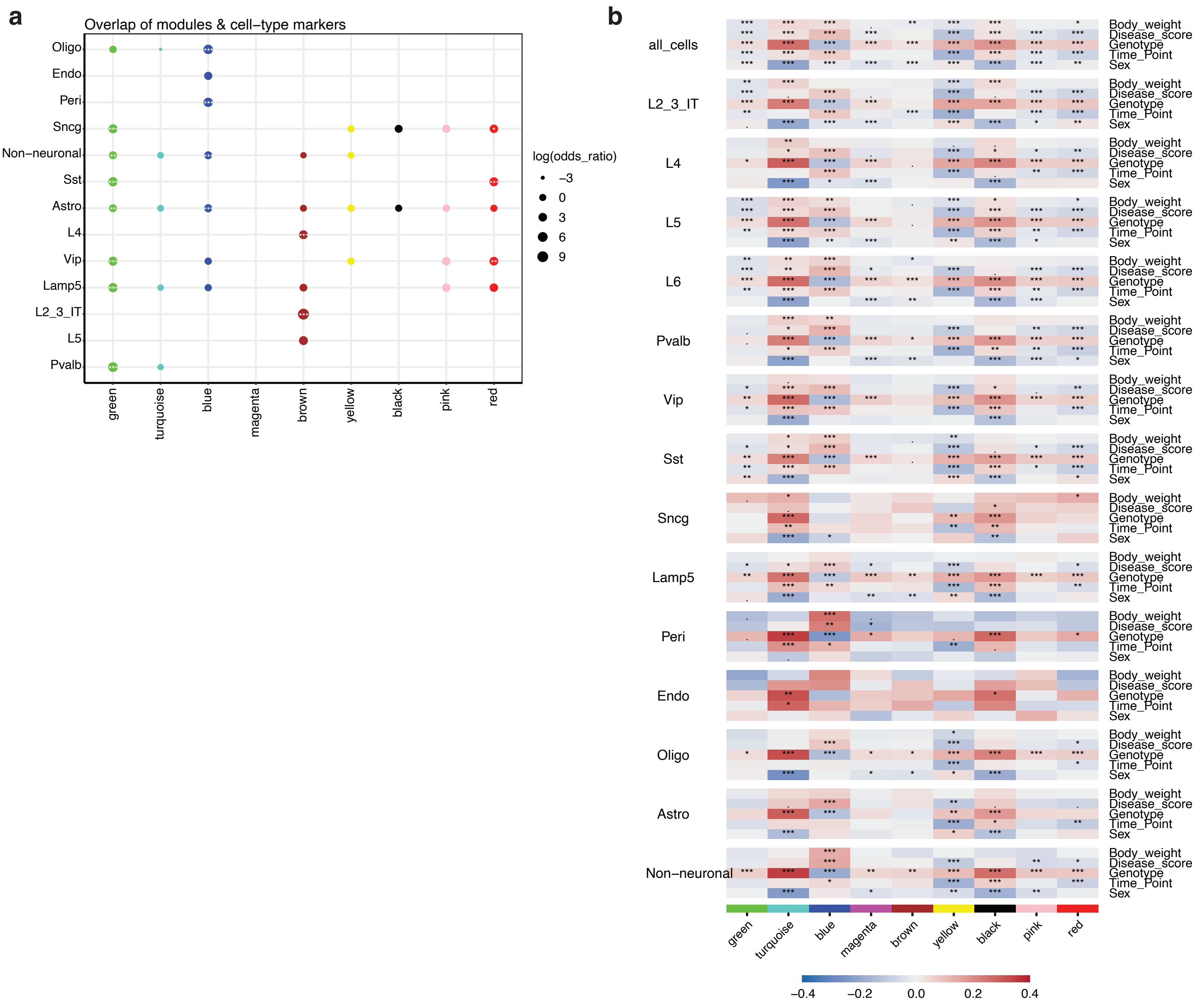

Supplementary fig.8: Top 10 hdWGCNA module KEGG terms

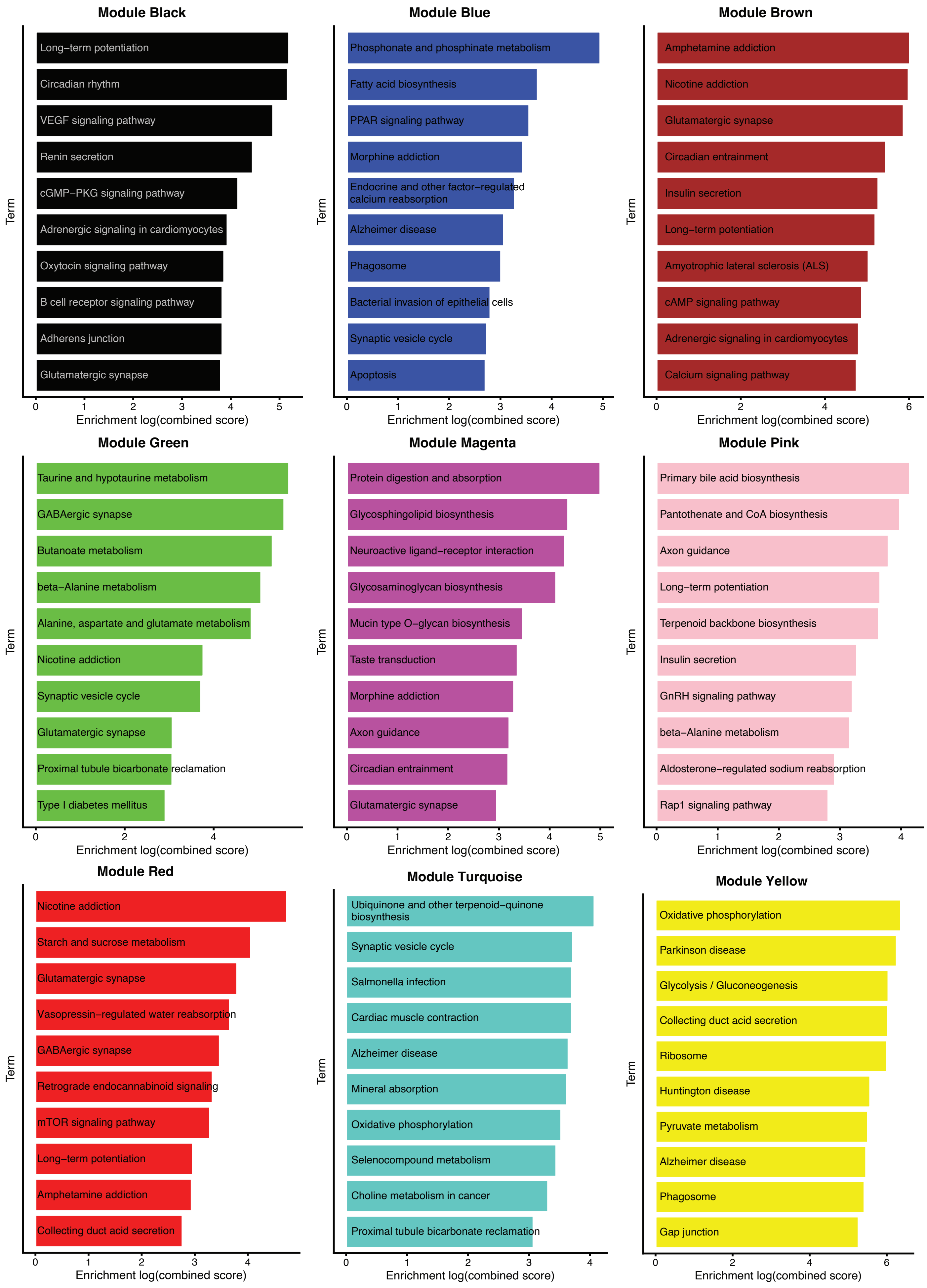

Supplementary fig.9: Bar graphs showing the ratio of WT *Mecp2* and Mutant *Mecp2* expressing cells

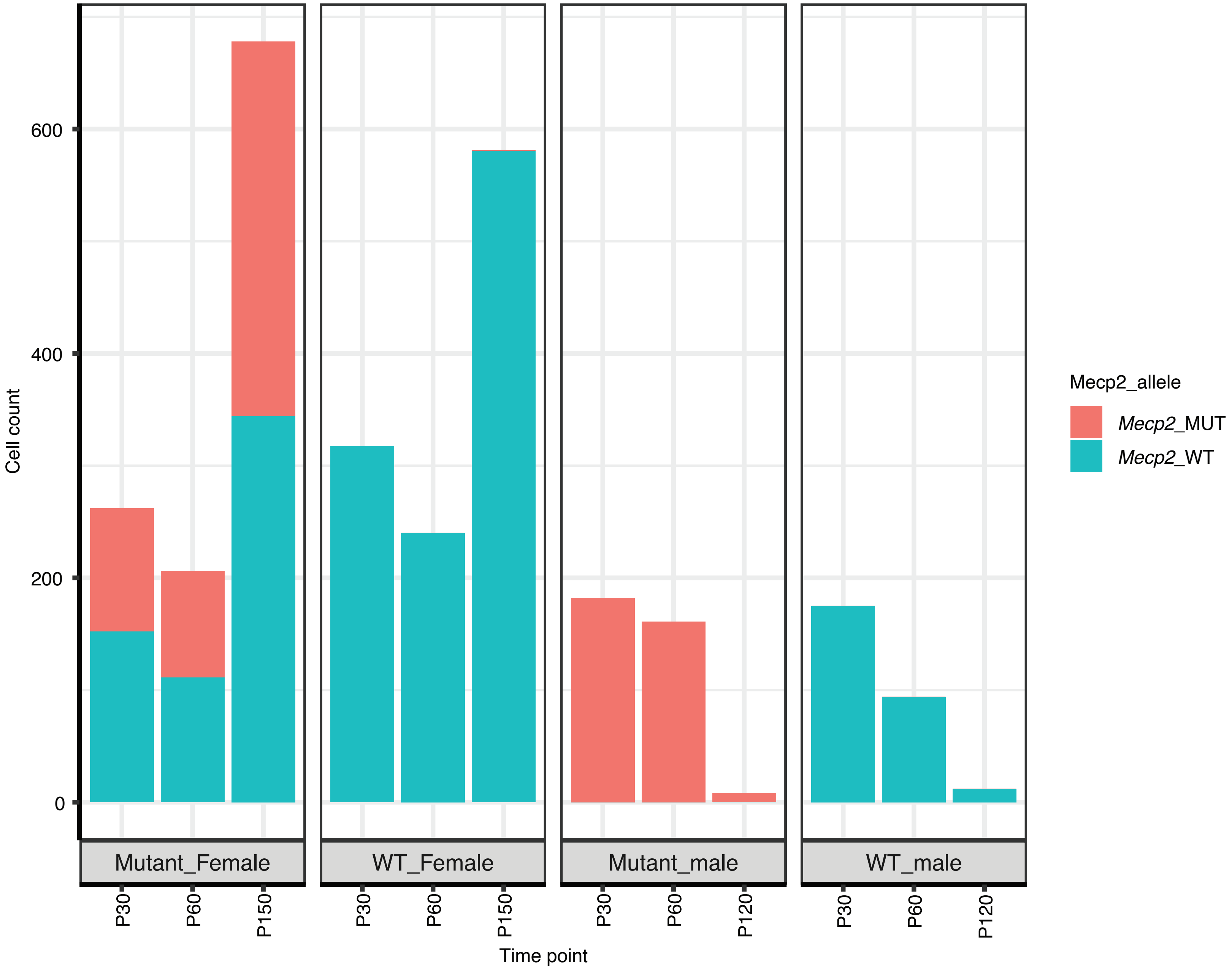

Supplementary fig.10: Differentially expressed genes comparing MUT cells from mosaic females and WT cells from WT females over time in Glutamatergic and GABAergic neurons. As shown in Experiment #4 (Fig. 1d), we compared MUT cells from Mecp2e1-/+ female with WT cells from Mecp2e1+/+ glutamatergic and GABAergic neurons longitudinally

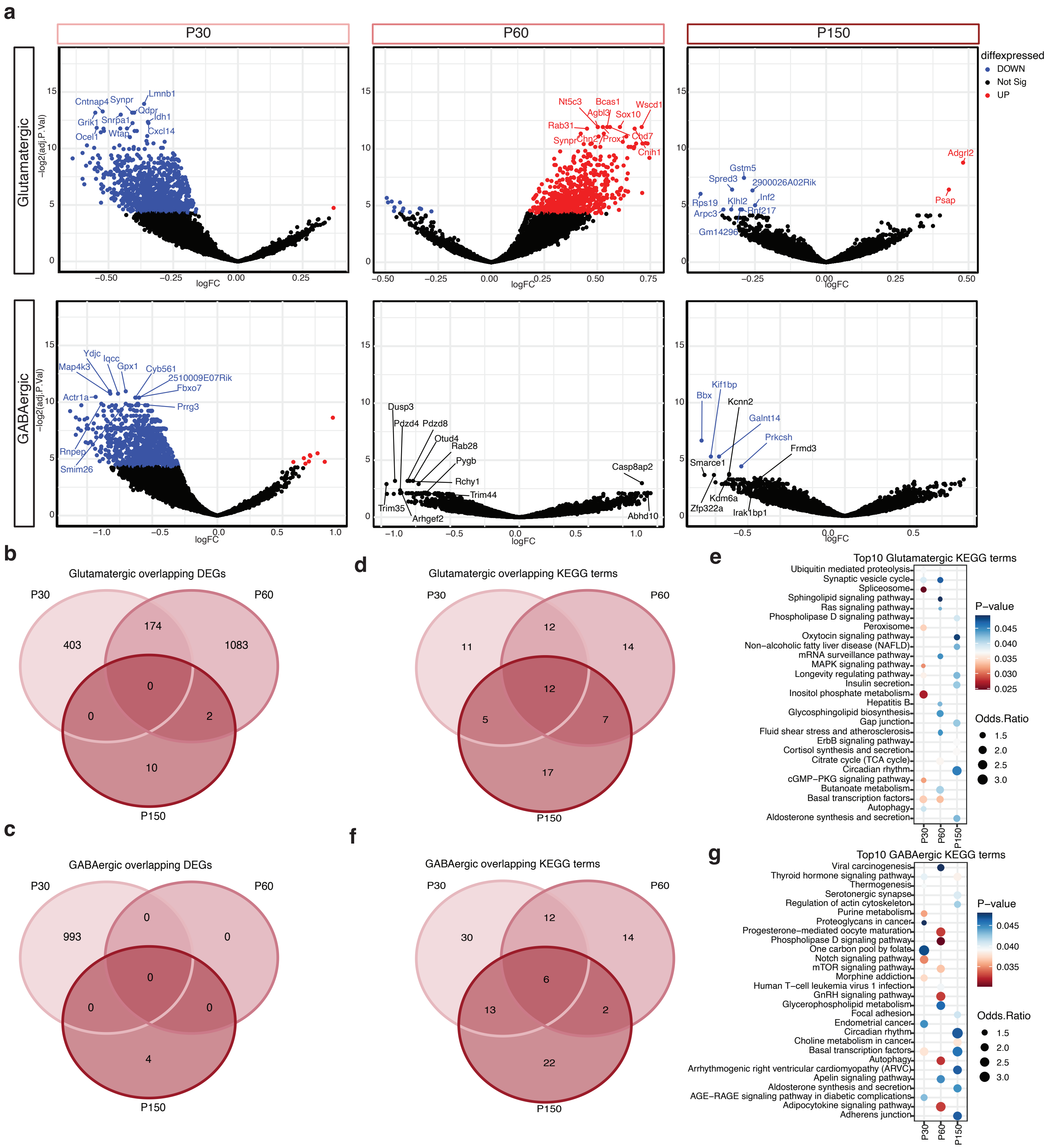

Supplementary fig.11: Overlap of KEGG terms from experiment 3 and experiment 4 over time

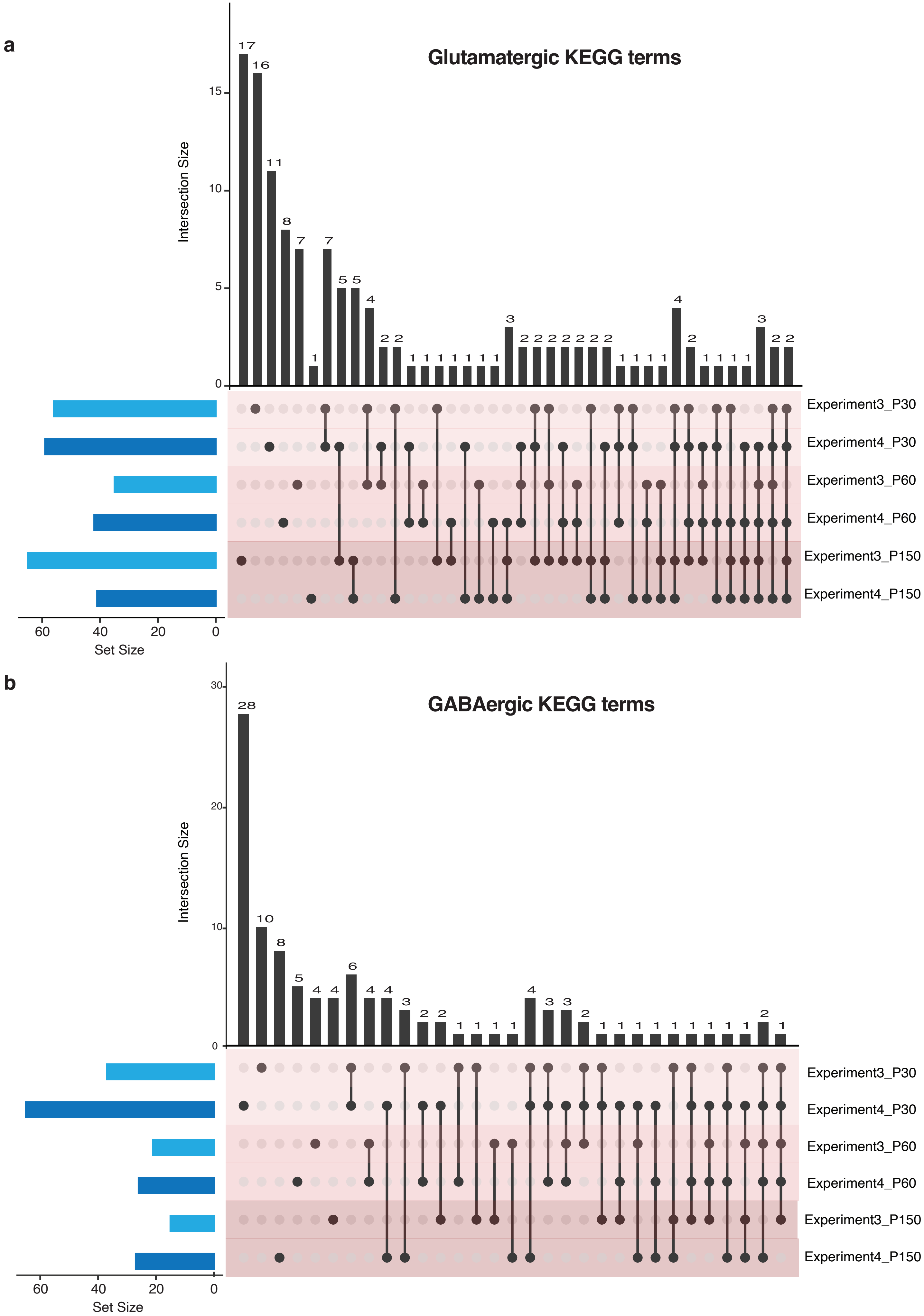

Supplementary fig.12: Differentially expressed genes and KEGG pathways comparing MUT and WT cells within *Mecp2e1*$^{-/+}$ female cortices over time in glutamatergic and GABAergic neurons

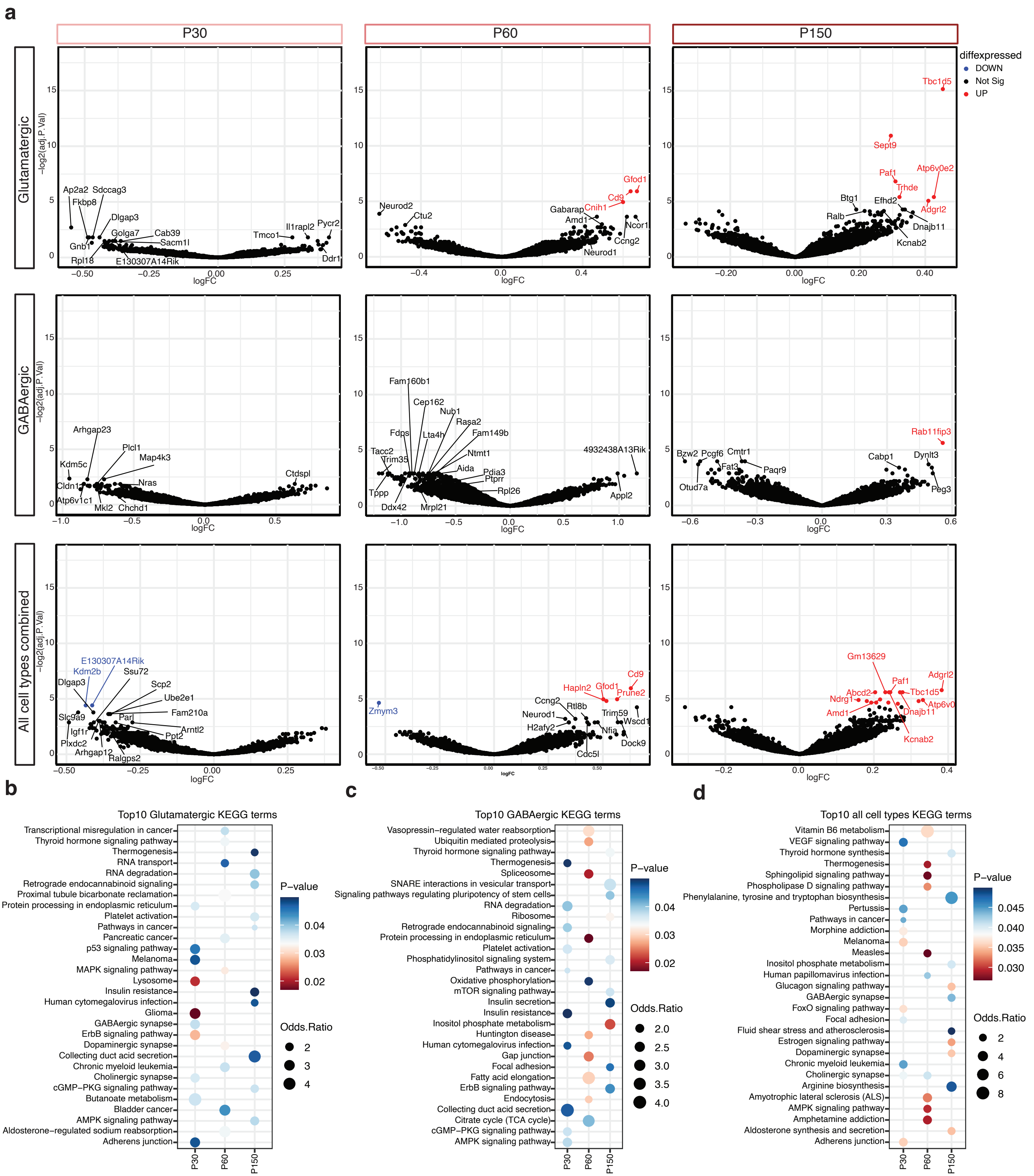

Supplement: Supplementary file 2 — Supplementary Information [file 42003_2024_6990_MOESM2_ESM.pdf]
