# Supplementary material for: Sex-specific single cell-level transcriptomic signatures of Rett syndrome disease progression
Source: Commun Biol. 2024 Oct 10;7:1292. doi: 10.1038/s42003-024-06990-0 (PMC11464704; doi:10.1038/s42003-024-06990-0)
Supplement: Supplementary file 3 — Description of Additional Supplementary Files [file 42003_2024_6990_MOESM3_ESM.pdf]

Description of Additional Supplementary Files

File name: Supplementary Data 1.
Description: Table containing all significant up and down regulated DEGs (LimmaVoomCC) from experiment 1 and 2. Table contains gene name, logFC, adjusted p-value, sex, cell type and time point information.

File name: Supplementary Data 2.
Description: Table containing all significant KEGG pathways from experiment 1 and 2. Table contains Term, overlap, odds ratio, adjusted p-value, Genes contained in the pathway, sex, cell type and metadata information.

File name: Supplementary Data 3.
Description: Table containing demographic and mutation information about human postmortem brain samples.
