# Supplementary material for: Sex-specific single cell-level transcriptomic signatures of Rett syndrome disease progression
Source: Commun Biol. 2024 Oct 10;7:1292. doi: 10.1038/s42003-024-06990-0 (PMC11464704; doi:10.1038/s42003-024-06990-0)
Supplement: Supplementary file 7 — Reporting summary [file 42003_2024_6990_MOESM7_ESM.pdf]

Corresponding author(s): COMMSBIO-24-2290-T

Last updated by author(s): Aug 1, 2024

Reporting Summary

Nature Portfolio wishes to improve the reproducibility of the work that we publish. This form provides structure for consistency and transparency in reporting. For further information on Nature Portfolio policies, see our Editorial Policies and the Editorial Policy Checklist.

Statistics

For all statistical analyses, confirm that the following items are present in the figure legend, table legend, main text, or Methods section.

n/a	Confirmed	
☐	☒	The exact sample size (*n*) for each experimental group/condition, given as a discrete number and unit of measurement
☐	☒	A statement on whether measurements were taken from distinct samples or whether the same sample was measured repeatedly
☐	☒	The statistical test(s) used AND whether they are one- or two-sided<br>*Only common tests should be described solely by name; describe more complex techniques in the Methods section.*
☐	☒	A description of all covariates tested
☐	☒	A description of any assumptions or corrections, such as tests of normality and adjustment for multiple comparisons
☐	☒	A full description of the statistical parameters including central tendency (e.g. means) or other basic estimates (e.g. regression coefficient) AND variation (e.g. standard deviation) or associated estimates of uncertainty (e.g. confidence intervals)
☐	☒	For null hypothesis testing, the test statistic (e.g. *F*, *t*, *r*) with confidence intervals, effect sizes, degrees of freedom and *P* value noted<br>*Give P values as exact values whenever suitable.*
☒	☐	For Bayesian analysis, information on the choice of priors and Markov chain Monte Carlo settings
☒	☐	For hierarchical and complex designs, identification of the appropriate level for tests and full reporting of outcomes
☐	☒	Estimates of effect sizes (e.g. Cohen's *d*, Pearson's *r*), indicating how they were calculated

Our web collection on statistics for biologists contains articles on many of the points above.

Software and code

Policy information about availability of computer code

Data collection	No software was used
Data analysis	Cellranger v.2.0.2 was used to aligned the mouse raw reads to mm10-1.2.0 reference genome and the human raw reads to GRCh38 human reference genome. Cell by gene count matrices were used to create a Seurat object using Seuraft_4.3.0.1 in R 4.2.2. DEG analysis tools used are edgeR 3.40.2, Limma 3.54.2, DeSeq2 1.38.3, and DEsingle 1.18.1. Kegg analysis was performed with enrichR 3.2. WGCNA analysis was performed via hdWGCNA 0.2.4. Cell parsing alignment uses abBlast 3.0, and BWA 0.7.17. Gene overlap statistics was performed via GeneOverlap 1.38.0. Plots were made via scCustomize 2.1.1 and ggplot2 3.4.2. Custom analysis pipeline is available in the github repository https://github.com/osmansharifi/snRNA-seq-pipeline.

For manuscripts utilizing custom algorithms or software that are central to the research but not yet described in published literature, software must be made available to editors and reviewers. We strongly encourage code deposition in a community repository (e.g. GitHub). See the Nature Portfolio guidelines for submitting code & software for further information.

Data

Policy information about availability of data

All manuscripts must include a data availability statement. This statement should provide the following information, where applicable:

- Accession codes, unique identifiers, or web links for publicly available datasets
- A description of any restrictions on data availability
- For clinical datasets or third party data, please ensure that the statement adheres to our policy

All data supporting the findings of this study are available within the paper and its Supplementary Information. snRNA-seq 5' data are deposited into the NCBI database, accession number PRJNA1142998.

Research involving human participants, their data, or biological material

Policy information about studies with human participants or human data. See also policy information about sex, gender (identity/presentation), and sexual orientation and race, ethnicity and racism.

Reporting on sex and gender	All human brain samples were female and biological sex was confirmed by the absence of Y chromosome reads. We have reported female sex of the samples in the manuscript.
Reporting on race, ethnicity, or other socially relevant groupings	Because Rett syndrome is a rare Mendelian disorder, race and ethnicity are not relevant and reported only for known cases.
Population characteristics	N/A
Recruitment	Human cerebral cortex samples were obtained from the National Institute of Child Health and Human Development (NICHD) Brain and Tissue Bank for Developmental Disorders at the University of Maryland.
Ethics oversight	Postmortem tissue is considered exempt from Human subject approval by NIH.

Note that full information on the approval of the study protocol must also be provided in the manuscript.

Field-specific reporting

Please select the one below that is the best fit for your research. If you are not sure, read the appropriate sections before making your selection.

☒ Life sciences ☐ Behavioural & social sciences ☐ Ecological, evolutionary & environmental sciences

For a reference copy of the document with all sections, see nature.com/documents/nr-reporting-summary-flat.pdf

Life sciences study design

All studies must disclose on these points even when the disclosure is negative.

Sample size	There are a total of 16 human and 28 mouse cortex samples. The human samples include 8 control and 8 RTT cortices, all female. Mouse samples include biological replicates for each sex, time point, and genotype.
Data exclusions	No data were excluded.
Replication	We benchmarked several DEG analysis tools for performance and chose limmaVoom. All experiments performed are reproducible across biological and technical replicates.
Randomization	Samples from each sex, genotype and time point were collected and processed randomly. All samples were sequenced together in order to minimize batch effects.
Blinding	The investigators were not blinded to the samples due to the genome-wide nature of the experiment. Control and disease samples were selected based on sex, genotype and time point. For all comparison analysis, disease and controls needed to be known in order to perform bioinformatic analyses.

Reporting for specific materials, systems and methods

We require information from authors about some types of materials, experimental systems and methods used in many studies. Here, indicate whether each material, system or method listed is relevant to your study. If you are not sure if a list item applies to your research, read the appropriate section before selecting a response.

Materials & experimental systems

n/a	Involved in the study
☒	☐ Antibodies
☒	☐ Eukaryotic cell lines
☒	☐ Palaeontology and archaeology
☐	☒ Animals and other organisms
☒	☐ Clinical data
☒	☐ Dual use research of concern
☒	☐ Plants

Methods

n/a	Involved in the study
☒	☐ ChIP-seq
☒	☐ Flow cytometry
☒	☐ MRI-based neuroimaging

Animals and other research organisms

Policy information about studies involving animals; ARRIVE guidelines recommended for reporting animal research, and Sex and Gender in Research

Laboratory animals	We used Mecp2e1 mutant mice on a C57Bl6/J background
Wild animals	*Provide details on animals observed in or captured in the field; report species and age where possible. Describe how animals were caught and transported and what happened to captive animals after the study (if killed, explain why and describe method; if released, say where and when) OR state that the study did not involve wild animals.*
Reporting on sex	Both sexes were investigated in mouse studies and all analyses were performed stratified by sex. Because Rett syndrome affects females, only females were investigated for human postmortem brain.
Field-collected samples	n/a
Ethics oversight	All animal procedures were approved by the UC Davis Institutional Animal Use and Care Committee (IACUC).

Note that full information on the approval of the study protocol must also be provided in the manuscript.

Plants

Seed stocks	n/a
Novel plant genotypes	n/a
Authentication	n/a
